# A Camera-Based Visual Sensor Pipeline for Fine-Grained Human Activity Recognition in Classroom Scenes

**DOI:** 10.3390/s26123666

**Published:** 2026-06-08

**Authors:** Cheng Sun, Danning Wu, Zihao Wu, Weibing Zhou, Jin Zhang

**Affiliations:** 1 School of Computer Science, Changsha University of Science and Technology, Changsha 410114, China; sun_ching@csust.edu.cn (C.S.); 24108031760@csust.edu.cn (D.W.); zihao_wu@csust.edu.cn (Z.W.); 25208031780@csust.edu.cn (W.Z.); 2 School of Information Science and Engineering, Hunan Normal University, Changsha 410081, China

**Keywords:** student behavior recognition, visual sensing, classroom video analysis, fine-grained action recognition, spatiotemporal modeling

## Abstract

Student behavior recognition in classroom environments is important for teaching quality assessment and intelligent education, yet it remains challenging due to dense student distributions, frequent occlusion, substantial scale variation, and the subtle nature of common classroom activities. To address these issues, this paper proposes RepYOLOv5-SF3D, a cascaded visual perception framework for fine-grained student behavior recognition in complex classroom scenes. The framework integrates a lightweight RepYOLOv5m detector with a dual-stream SlowFast-3D recognition branch, enabling automated inference from raw video input to behavior labels. To improve robustness in dense and occluded scenes, the front-end detector serves as a spatial-prior module, while a decoupled training strategy reduces the impact of localization instability on back-end spatiotemporal learning. In addition, two task-oriented modules are introduced in the recognition branch: the Spatiotemporal Depthwise-Separable 3D module (SDS3D) and the Normalization-Based Temporal Attention Mechanism (NTAM). Experimental results on a real classroom dataset show that RepYOLOv5-SF3D achieves a mean average precision (mAP) of 88.83%, outperforming the baseline SlowFast model by 3.36% and surpassing the existing LSTC method by 2.05%, while maintaining a front-end inference latency of 12.5 ms per frame and a total model size of 151.46 MB. These results demonstrate a favorable balance between fine-grained recognition accuracy and edge-deployment efficiency in practical classroom visual sensing.

## 1. Introduction

Student behavior analysis in classroom environments is an important basis for teaching quality evaluation and an essential component of intelligent education systems. Its purpose is to analyze classroom observation data so as to objectively reflect students’learning engagement and their immediate responses to instructional activities. Compared with traditional evaluation approaches based on manual observation, post-class summarization, or limited sampling, vision-based student behavior analysis enables continuous and fine-grained observation across longer time spans and larger student populations, thereby improving the objectivity, quantifiability, and timeliness of instructional assessment [1]. Although contact-based physiological sensing and wearable devices can provide partial information on student states, their high intrusiveness, deployment cost, and potential interference with classroom activities make them difficult to apply on a large scale in real teaching scenarios. In contrast, surveillance cameras that are already widely deployed in classrooms can serve as non-contact visual sensors to continuously capture the spatial distribution and behavioral dynamics of student groups with relatively low interference, thus providing a practical data source for classroom behavior perception [2]. With the increasing availability of such visual data, classroom behavior analysis has gradually evolved from isolated action recognition toward a more comprehensive visual perception task in real educational environments. Recent studies have further confirmed the growing interest in classroom-oriented student behavior detection and recognition, especially under practical instructional settings involving multiple students, cluttered backgrounds, and severe occlusion [3].

However, real classroom scenes remain highly challenging for vision-based analysis. Unlike ideal observation settings, classrooms are typically characterized by dense targets, frequent occlusion, and substantial scale variation. In practical teaching environments, students are often arranged in compact seating layouts, where desk obstruction and inter-row overlap can easily lead to missed detection or inaccurate localization in the front-end stage. In addition, common classroom behaviors, such as looking up, looking down, and speaking, usually involve subtle motion patterns, high inter-class similarity, and only slight local appearance differences, which make reliable recognition difficult when relying solely on static visual information [4]. Existing spatiotemporal modeling methods, including conventional 3D convolutional networks and generic SlowFast architectures, are capable of jointly modeling spatial and temporal cues, but they are mainly designed for large-scale actions in open scenarios [5]. More recently, transformer-based video architectures have also demonstrated strong spatiotemporal modeling capability, indicating that video understanding has evolved beyond conventional convolution-based designs [6]. When applied to classroom behavior recognition, these methods often suffer from relatively high computational cost, insufficient sensitivity to subtle behavioral dynamics, and limited ability to emphasize informative temporal cues [7]. More importantly, in cascaded recognition frameworks, the quality of back-end behavior classification is highly dependent on the stability of front-end localization. Once candidate regions become inaccurate or inconsistent, recognition errors may accumulate across stages and degrade the overall system performance. Therefore, the key problem is not merely the local improvement of a single module, but the construction of a coordinated perception framework that can jointly address dense-target localization and fine-grained spatiotemporal representation while maintaining a reasonable balance between accuracy and efficiency.

To address these issues, we propose RepYOLOv5-SF3D, a cascaded framework for classroom behavior recognition in complex scenes. The proposed framework combines a RepYOLOv5m-based student detector with a SlowFast-3D recognition branch (SF3D), thereby enabling efficient recognition of student behaviors in dense classroom environments. At the framework level, the method supports automated inference from raw video input to behavior label output, while adopting a decoupled training strategy to reduce the interference of front-end detector fluctuations with back-end spatiotemporal feature learning. Specifically, a lightweight RepYOLOv5m detector is introduced as a front-end spatial-prior module to improve detection recall under dense occlusion while preserving computational efficiency for edge deployment. On this basis, the back-end SF3D branch is constructed on a dual-stream spatiotemporal architecture and further enhanced by two dedicated modules, namely the Spatiotemporal Depthwise-Separable 3D module (SDS3D) and the Normalization-Based Temporal Attention Mechanism (NTAM). SDS3D improves the efficiency and discriminative capacity of spatiotemporal representation by alleviating the redundancy of conventional 3D convolutions, whereas NTAM reuses normalization scale factors to realize parameter-free temporal emphasis, thereby enhancing the perception of subtle behavioral dynamics without introducing additional learnable parameters. Through the collaboration of front-end localization and back-end recognition, the proposed framework aims to improve the robustness and efficiency of classroom behavior analysis under dense and occluded conditions:

- An efficient cascaded framework for classroom behavior analysis is established. By integrating lightweight student localization with dual-stream spatiotemporal recognition, the proposed method improves the overall robustness and efficiency of behavior perception in classroom scenes involving dense targets and complex interactions.
- Two task-oriented feature enhancement modules are designed for subtle classroom behaviors. SDS3D improves spatiotemporal representation by introducing depthwise-separable decoupling into 3D modeling, whereas NTAM achieves parameter-free temporal emphasis through the reuse of normalization scale factors, thereby enhancing discrimination among behaviors with small motion amplitudes and high visual similarity.
- The proposed framework exhibits favorable potential for practical deployment on edge devices. Experimental results on real classroom datasets indicate that RepYOLOv5-SF3D attains a competitive balance between recognition accuracy and inference speed under dense occlusion, while also showing advantages in model size and latency relative to mainstream spatiotemporal networks.

The remainder of this paper is organized as follows. Section 2 reviews related work on spatiotemporal modeling for action recognition and attention mechanisms in video analysis. Section 3 details the proposed RepYOLOv5-SF3D framework, including the front-end RepYOLOv5m detector, the decoupled training strategy, and the design of the SDS3D and NTAM modules. Section 4 presents the experimental setup, dataset characteristics, ablation studies, and comparative evaluations against mainstream behavior recognition methods. Section 5 discusses the practical implications of the proposed method, its lightweight design advantages, and directions for future work. Finally, Section 6 concludes the paper.

## 2. Related Work

### 2.1. Spatiotemporal Modeling for Video-Based Human Action Recognition

The core of video-based human action recognition lies in joint spatiotemporal modeling. Unlike static image classification, video analysis must not only identify spatial targets but also capture their dynamic evolution over time [8]. To address this requirement, spatiotemporal modeling has progressed from 2D convolution with temporal aggregation, to direct modeling with standard 3D convolution, and further to dual-stream collaborative architectures based on spatiotemporal factorization. Early methods focused primarily on aggregating frame-level image features over time. Later work shifted toward explicit extraction of joint spatiotemporal representations. More recent studies have concentrated on balancing recognition accuracy and inference efficiency while preserving model discrimination.

Early video action recognition methods generally followed a two-stage strategy. A 2D convolutional network first extracted spatial features from individual frames, after which temporal information was integrated through temporal pooling, frame sampling, or recurrent modeling. The main advantage of this line of work is its relatively low training cost and its ability to inherit the representational strength of image classification models, which gives it practical value when data scale or computational resources are limited [9]. However, temporal modeling in such methods remains structurally separated from spatial representation and is introduced only after frame-level feature extraction. This limits their ability to capture continuous local dynamics during action execution. In classroom scenarios with complex visual conditions, this limitation becomes more pronounced. When actions are subtle or severe occlusion occurs, frame-level features are often insufficient to support robust decision boundaries, and later temporal aggregation cannot compensate for information lost at the perception stage.

To overcome the weak joint modeling capability of two-stage approaches, researchers introduced 3D convolution into human action recognition. Unlike post hoc temporal aggregation, 3D convolution extracts spatial structure and motion information simultaneously within local spatiotemporal neighborhoods, improving the completeness of action modeling. Along this line, factorizing standard 3D convolution has proved effective for reducing computational redundancy while stabilizing optimization. The R(2+1)D network proposed by Tran et al. factorizes standard 3D convolution into spatial and temporal convolutions, strengthening nonlinear representation while improving the training process [10]. The P3D residual network proposed by Qiu et al. adopts a pseudo-3D factorization scheme, combining spatial and temporal convolutions to improve spatiotemporal representation under controlled parameter complexity [11]. Subsequent research developed in two directions. One direction reduced redundant computation in 3D convolution through channel-separated and depthwise-separable designs [12]. The other improved efficient video modeling through more flexible temporal aggregation strategies [13]. These developments indicate that the central issue in spatiotemporal modeling is how to reduce computational cost without weakening discrimination.

More recently, dual-stream spatiotemporal networks have further advanced video action recognition. Compared with single-path architectures, dual-stream designs assign different temporal scales to different pathways, producing more structured video representations. One of the most representative frameworks is SlowFast [14], which consists of two pathways with different sampling rates: a slow pathway that models stable semantics and spatial structure at a low frame rate, and a fast pathway that captures short-term motion variations at a higher frame rate. This design establishes an effective structural balance between semantic stability and motion sensitivity and has achieved strong performance on general video action recognition benchmarks. For classroom scenarios, this formulation is particularly suitable. Semantic information such as student targets and seat regions remains relatively stable, whereas actions such as hand raising, head turning, and speaking are mainly expressed through short-term local variations. This matches the dual-stream principle of separately modeling stable semantics and rapid dynamics. Accordingly, dual-stream spatiotemporal modeling provides a strong foundation for fine-grained behavior recognition in classroom videos. At the same time, recent work has increasingly emphasized efficient video understanding, showing that the trade-off between accuracy and computational cost has become a central concern in practical video analysis systems [15].

However, existing spatiotemporal modeling methods, especially standard stacked 3D convolution structures and generic SlowFast architectures, still face two limitations in classroom scenarios. First, classroom behaviors are subtle and highly similar, and general-purpose spatiotemporal models, although capable of joint modeling, still lack sufficient sensitivity to such fine motion patterns. Second, these models are typically designed for general action recognition in open environments and often incur substantial computational cost, which limits real-time inference and edge-side deployment under classroom resource constraints. Therefore, in complex classroom visual sensing scenarios, the central problem of spatiotemporal modeling is how to strengthen fine-grained action discrimination while reducing convolutional redundancy, and how to further improve temporal focus on informative motion cues.

### 2.2. Attention Mechanisms in Video Action Analysis

In fine-grained action recognition, the backbone network determines the basic capacity for feature extraction, whereas directing the model toward discriminative spatiotemporal cues forms another major route to stronger recognition. For this reason, attention mechanisms have been widely incorporated into video analysis pipelines. Their central role is to adaptively reshape feature importance so that limited computation is concentrated on the most discriminative local dynamics.

Representative studies include the Non-local module based on global dependency modeling [16], as well as feature recalibration structures such as SENet [17] and CBAM [18]. The former explicitly models long-range dependencies by computing pairwise correlations across spatiotemporal positions, compensating for the limited receptive field of local convolution. The latter dynamically recalibrates channel or spatial features through additional fully connected or convolutional branches, allowing the network to amplify more discriminative feature responses. Some studies have further extended attention mechanisms to the temporal dimension, aiming to identify key segments in long video sequences that better characterize the essence of an action and thereby strengthen dynamic modeling [19,20]. Beyond explicit temporal attention, recent studies have also explored more efficient temporal modeling strategies, such as learnable temporal alignment, to capture motion cues without relying solely on conventional temporal attention operations [21]. These methods have achieved strong results on general action recognition benchmarks, confirming the effectiveness of attention mechanisms in improving video representation quality.

Despite their strong performance on general video benchmarks, these mechanisms expose a clear domain-adaptation gap in real classroom videos. Classroom scenes often exhibit a low-signal-to-noise-ratio video stream filled with redundant static background content and meaningless local micro-jitters. Under such conditions, conventional attention modules struggle to isolate background noise precisely along the temporal dimension, which weakens their ability to capture high-frequency key dynamics [22]. In addition, mainstream solutions rely heavily on explicit weight-generation branches. Whether implemented through multilayer perceptrons or complex parameterized mapping layers, these designs introduce extra parameters and structural redundancy that directly conflict with the lightweight deployment requirements of visual perception systems on front-end edge devices. Therefore, a key theoretical and engineering challenge in fine-grained behavior recognition is how to achieve adaptive weighting of critical temporal segments and effective background suppression in a parameter-free manner. In addition, recent fine-grained action recognition studies have suggested that pose-guided representations can further improve the discrimination of subtle motions, which may also be beneficial for classroom scenarios involving slight head, hand, and upper-body movements [23].

The preceding analysis reveals a clear gap in existing attention-based approaches when applied to fine-grained classroom behavior recognition under edge-deployment constraints. First, the majority of temporal attention modules—whether based on non-local operations, self-attention mechanisms, or learnable temporal shifts—introduce explicit parameterized branches to generate frame-wise or segment-wise importance weights, while effective on large-scale benchmarks, these auxiliary structures increase the overall parameter count and memory access overhead, which directly conflicts with the stringent latency requirements of multi-stream classroom surveillance on edge devices. Second, in low-signal-to-noise classroom videos where behavioral cues are subtle and background content dominates, conventional attention mechanisms often lack the precision to isolate task-relevant dynamics from static scene redundancy and meaningless micro-jitters, thereby diluting the model’s sensitivity to fine-grained action boundaries. We hypothesize that a parameter-free temporal calibration mechanism—derived directly from the inherent scale statistics of batch normalization layers rather than from auxiliary learnable branches—can achieve effective temporal emphasis for fine-grained classroom behaviors while maintaining strict computational parsimony. Furthermore, we posit that coupling such a mechanism with a structurally diverse set of factorized spatiotemporal convolutions will jointly enhance the discrimination of low-amplitude, occlusion-prone actions without inflating the parameter footprint of the recognition backbone.

### 2.3. Lightweight Spatiotemporal Modeling for Edge Devices

With the growing demand for real-time video analytics in resource-constrained environments, substantial research efforts have been devoted to developing lightweight spatiotemporal models suitable for edge deployment. Early work in this direction explored depthwise separable 3D convolutions and factorized (2+1)D architectures to reduce the computational redundancy of standard 3D convolutions while preserving joint spatiotemporal modeling capacity [10,11]. More recent studies have further pursued lightweight designs explicitly tailored for edge computing. For instance, SlimSTAD proposed a dual cross-modal encoder for efficient spatiotemporal action detection, achieving both high accuracy and low latency for sensor-based applications [24]. YOWOv3 introduced a lightweight spatiotemporal joint network that employs a spatiotemporal shift module containing only 2D convolutions, mitigating potential compatibility issues with 3D convolution operators on edge hardware while maintaining real-time performance [25]. In the context of video transformers, VETA-CLIP adopted lightweight adapters and efficient spatiotemporal attention mechanisms to achieve competitive accuracy with substantially reduced computational overhead [26]. The Adaptive Sparse Multimodal Transformer (ASMT) further reduced inference latency by dynamically selecting informative tokens via lightweight gating modules, achieving up to 63% reduction in FLOPs on edge-oriented hardware [27]. EdgeOAR addressed online action recognition under stringent latency constraints by incorporating early-exit mechanisms and lightweight feature enhancement modules, enabling real-time feedback on resource-constrained devices [28]. However, these methods often target general action recognition benchmarks or rely on multimodal sensor fusion. A dedicated lightweight cascaded framework that jointly optimizes dense student detection and fine-grained spatiotemporal behavior recognition for edge deployment in classroom environments remains underexplored, motivating the design of RepYOLOv5-SF3D in this work.

## 3. Methods

### 3.1. A Visual Perception Pipeline for Complex Classroom Scenarios

**Figure 1:**
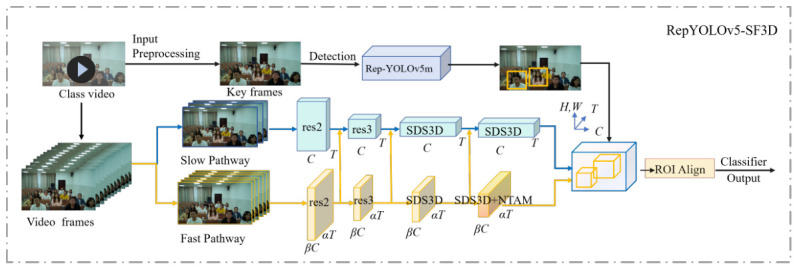
Overall architecture of RepYOLOv5-SF3D.

For video-based behavior detection in real classroom environments, system performance is constrained not only by the representational capacity of the back-end recognition network, but also by how the computational budget is allocated across the entire pipeline [29]. In classroom scenes with dense student distributions, frequent occlusion, and continuous inference demands, excessive computation in the front-end detector can become a major latency bottleneck for subsequent spatiotemporal recognition [30]. To address this issue, we develop RepYOLOv5-SF3D, a cascaded detection–recognition framework that integrates lightweight spatial-prior extraction, local spatiotemporal alignment, and dual-stream behavior recognition. The overall architecture is shown in Figure 1.

At the input stage, key frames are sampled from raw classroom videos at 30 fps and fed into the front-end detector to generate student bounding boxes. Subsequent behavior recognition is then performed on the detected regions rather than on the full frame, which reduces background interference and improves downstream inference efficiency.

The front end adopts RepYOLOv5m, derived from RepGhost, as a hardware-aware student detector and spatial-prior module [31]. The objective of this design is to reduce front-end latency rather than to increase detector complexity. During training, the network retains multi-branch convolutional and normalization structures to enhance feature learning. During inference, these branches are re-parameterized into a single equivalent convolution, thereby reducing memory access and computational overhead. In addition, RepGhost replaces explicit feature concatenation with additive implicit feature reuse, which further decreases memory-copy cost during inference. For dense classroom scenes, this design reduces the computational burden of multi-student detection while maintaining high recall, thus reserving more computational resources for back-end behavior recognition.

Once the front-end detector outputs 2D student bounding boxes on the sampled key frame, the system constructs a short-term spatiotemporal proposal for each detected student based on the key-frame box. Specifically, the proposed framework is key-frame-centered rather than tracking-based. In other words, we do not introduce a separate multi-object tracking or explicit temporal linking module to associate student identities across frames. Instead, the detected box on the key frame is directly extended to a local temporal window centered at that frame, thereby forming a student-centered cuboid proposal in the feature space. 3D RoI Align is then applied to this cuboid to extract locally aligned spatiotemporal features for the downstream recognition branch. As a result, the back-end network operates on local student-centered spatiotemporal regions rather than on redundant full-scene classroom videos.

Formally, let the detected student bounding box on the key frame $t_{0}$ be denoted by

(1) $$b_{t_{0}}=\left(x_{1},y_{1},x_{2},y_{2}\right),$$

where $\left(x_{1},y_{1}\right)$ and $\left(x_{2},y_{2}\right)$ represent the coordinates of the top-left and bottom-right corners, respectively. For a short temporal clip, we define the local temporal window as

(2) $$T=\{t_{0}-\Delta ,\ldots ,t_{0}+\Delta \},$$

where Δ is the temporal radius. The spatial proposal is then extended along the temporal dimension by reusing the same box coordinates for all frames within the temporal window:

(3) $$b_{t}=b_{t_{0}},\;\forall t\in T.$$

Accordingly, the spatiotemporal cuboid proposal is constructed as

(4) $$B=\{b_{t}|t\in T\}.$$

Given the backbone feature volume $F\in R^{T\times H\times W\times C}$, 3D RoI Align is applied on *B* to extract a fixed-size local spatiotemporal feature tensor, which is subsequently fed into the back-end recognition network. The overall key-frame-centered local spatiotemporal alignment procedure is summarized in Algorithm 1.
**Algorithm 1** Key-Frame-Centered Local Spatiotemporal Alignment**Require:** 
Video clip *V*, key-frame index $t_{0}$, student detector *D*, temporal radius Δ**Ensure:** 
Local spatiotemporal proposals for student behavior recognition1:Sample the key frame $I_{t_{0}}$ from *V*2:Apply *D* on $I_{t_{0}}$ to obtain detected student boxes $B_{t_{0}}={\left\{b_{t_{0}}^{i}\right\}}_{i=1}^{N}$3:**for** each detected box $b_{t_{0}}^{i}\in B_{t_{0}}$ **do**4:   Define a local temporal window $T=\{t_{0}-\Delta ,\ldots ,t_{0}+\Delta \}$5:   **for** each t∈T **do**6:     Assign $b_{t}^{i}\leftarrow b_{t_{0}}^{i}$7:   **end for**8:   Form the cuboid proposal $B^{i}=\left\{b_{t}^{i}|t\in T\right\}$9:   Perform 3D RoI Align on $B^{i}$ to extract the fixed-size feature tensor $F_{\mathrm{roi}}^{i}$10:**end for**11:Feed ${\left\{F_{\mathrm{roi}}^{i}\right\}}_{i=1}^{N}$ into the SlowFast-3D recognition branch12:Output the predicted behavior label for each detected student

It should be emphasized that this design does not perform explicit trajectory estimation or long-term identity tracking under prolonged heavy occlusion. Instead, robustness to moderate occlusion mainly comes from two aspects of the framework: first, the front-end detector provides relatively reliable spatial priors in dense classroom scenes; second, the back-end spatiotemporal recognition branch aggregates short-term dynamic information within the local temporal window, which alleviates moderate localization instability. This design improves pipeline simplicity and computational efficiency while remaining suitable for short-term classroom behavior perception.

After aligned local spatiotemporal features are obtained, the system generates two temporal input streams under a dual-stream sampling strategy and routes them to the SlowFast-3D recognition branch (SF3D). The Slow pathway models relatively stable semantic content and spatial structure at a lower temporal sampling density, whereas the Fast pathway preserves local motion variations at a higher temporal sampling density. Lateral feature interactions are performed at multiple stages between the two pathways, allowing semantic and dynamic information to be fused within a unified framework. The fused features are then processed by the pooling layer and classification head, and the final behavior category of each detected student is produced.

Through this design, RepYOLOv5-SF3D forms a unified perception pipeline consisting of key-frame localization, key-frame-guided local spatiotemporal alignment, and dual-stream behavior recognition, thereby providing an efficient system-level solution for student behavior analysis in dense classroom environments.

### 3.2. The SDS3D Network for Fine-Grained Classroom Behaviors

Classroom behaviors exhibit two salient characteristics. First, motion amplitudes are usually small, and pronounced movement is relatively uncommon. Second, different behavior classes are often highly similar in local appearance. For example, looking down while writing, briefly lying on the desk, and slight head turning may differ only in subtle posture cues and short-term motion patterns. Under such conditions, standard 3D convolution, although capable of joint spatial–temporal modeling, tends to produce relatively homogeneous spatiotemporal response patterns because of its fixed convolutional form [8]. This can weaken class discrimination when the model is required to distinguish highly similar behaviors.

**Figure 2:**
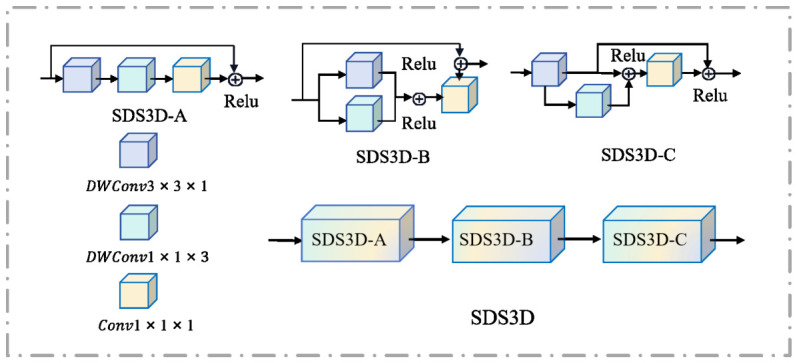
Structures of the SDS3D module.

To address this problem, we replace standard 3D convolution with a factorized (2+1)D spatiotemporal representation [10], explicitly separating spatial appearance modeling from temporal dynamics modeling. On this basis, we design the Spatiotemporal Depthwise-Separable 3D module (SDS3D), which constructs diverse spatiotemporal response paths through three structural variants. This design improves the network’s ability to characterize fine-grained behavior differences and structurally reduces the representational redundancy of conventional 3D convolutions, while strengthening the separate modeling of local structure and motion variation. In the present work, this efficiency motivation is discussed mainly from the perspective of factorized spatiotemporal modeling and overall model complexity, rather than from a dedicated hardware-level profiling of each SDS3D variant. The three substructures of SDS3D are shown in Figure 2.

SDS3D-A: Serial Residual Pattern

This variant adopts a serial path consisting of spatial convolution followed by temporal convolution, with the result fused with the input feature through a residual connection. Temporal variation is extracted on top of stabilized spatial semantics, making this pattern suitable for behaviors with relatively stable localization and relatively slow motion changes, such as sustained looking down, slow head lifting, or gradual turning. The connection pattern is described by Equations (5) and (6), Where *I* denotes identity mapping, Conv denotes the 1×1×1 convolution kernel, $x_{t}$ and $x_{t+1}$ denote the input and output features, respectively.

(5) $$\left(I+T\cdot S\right)\cdot x_{t}:=x_{t}+\mathrm{Conv}\left(T(S\left(\mathrm{Conv}\left(x_{t}\right)\right))\right)=x_{t+1}$$

(6) T=DWConv(3×3×1),S=DWConv(1×1×3)

SDS3D-B: Parallel Complementary Pattern

This variant performs spatial convolution and temporal convolution in parallel, allowing appearance variation and motion variation to be learned independently from the same input before being fused at the output. Compared with the serial structure, the parallel design weakens the strong coupling between spatial and temporal features. It is therefore more suitable for behaviors in which spatial deformation and temporal change are not tightly synchronized, such as brief hand raising or rapid head turning. The computation is given in Equation (7).

(7) $$\left(I+T+S\right)\cdot x_{t}:=x_{t}+\mathrm{Conv}\left(S\left(\mathrm{Conv}\left(x_{t}\right)\right)+T\left(\mathrm{Conv}\left(x_{t}\right)\right)\right)=x_{t+1}$$

SDS3D-C: Hybrid Cascaded Pattern

This variant introduces hierarchical coupling between spatial and temporal information through a double-residual mechanism. The output of spatial convolution is first fed back into the main feature stream, after which temporal convolution is performed in the updated semantic space, and a second residual connection produces the final output. This hybrid cascaded design preserves both direct interaction and indirect propagation between spatial and temporal information, allowing more discriminative representation to form at higher semantic levels. The two-stage residual computation is given in Equations (8) and (9).

(8) $$\left(I+T+T\cdot S\right)\cdot x_{t}:=x_{t}+\mathrm{Conv}\left(S^{\prime }\left(x_{t}\right)+T\left(S^{\prime }\left(x_{t}\right)\right)\right)=x_{t+1}$$

(9) $$S^{\prime }\left(x_{t}\right)=S\left(\mathrm{Conv}\left(x_{t}\right)\right)$$

From a structural perspective, SDS3D-A emphasizes progressive spatiotemporal modeling, SDS3D-B emphasizes complementary responses between spatial and temporal cues, and SDS3D-C emphasizes hierarchical interaction between the two. In the final implementation, as illustrated in the lower-right part of Figure 2, SDS3D-A, SDS3D-B, and SDS3D-C are cascaded to form one complete SDS3D module. This design enables the network to construct richer spatiotemporal receptive fields and more diverse response paths within a unified block, thereby improving discrimination among highly similar classroom behaviors with small motion amplitudes.

Considering the hierarchical characteristics of the SlowFast backbone, the complete SDS3D module is deployed only in the deeper res4 and res5 stages while the shallow layers remain unchanged. Specifically, two SDS3D modules are inserted into res4 and one SDS3D module is inserted into res5. The rationale is that introducing temporally modeled convolution kernels too early in shallow layers may reduce accuracy, whereas deeper layers provide broader spatial receptive fields and stronger semantic representations, making them more suitable for targeted spatiotemporal difference modeling. This arrangement allows high-level features with a wider global view to support higher-quality spatiotemporal representation for fine-grained classroom behaviors.

### 3.3. The NTAM Based on Normalization Factors

**Figure 3:**
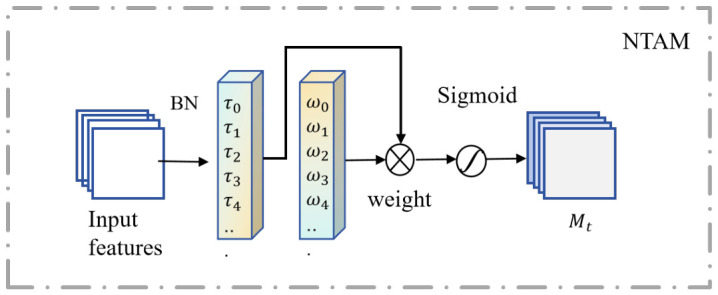
Structure of the NTAM.

Temporal cues form the core basis for distinguishing fine-grained actions in video behavior recognition. Although the Fast pathway preserves subtle motion through high-frame-rate sampling, this high-frequency sensing mechanism also introduces a large number of redundant static frames and invalid local micro-jitters. To strengthen the response to key dynamic segments without incurring substantial computational overhead, we incorporate a Normalization-Based Temporal Attention Mechanism (NTAM) into the proposed framework. The design of NTAM is inspired by the normalization-based attention strategy of Liu et al. [32], while being adapted here to temporal dynamic emphasis for fine-grained classroom behavior recognition. Its structure is shown in Figure 3.

Unlike conventional attention designs such as SENet, which rely heavily on additional fully connected layers or convolutional branches for weight generation [17], NTAM adopts a compact architecture for parameter-efficient temporal recalibration. Similar to the normalization-based reweighting idea in NAM [32], NTAM reuses the statistics and affine parameters produced by the Batch Normalization (BN) [33] operation. In our framework, however, this mechanism is specifically introduced after the temporal convolution branch of SDS3D, so as to emphasize informative temporal responses in classroom video clips while avoiding substantial additional parameter overhead. Specifically, NTAM first applies normalization to the input feature along the temporal dimension. This operation centers and scales the feature distribution using the mean and variance of the current mini-batch, thereby establishing a normalized reference for temporal fluctuation. The normalization process can be written as Equation (10).

(10) $$B_{t}=\mathrm{BN}\left(B_{\mathrm{input}}\right)=\gamma_{i}\frac{B_{\mathrm{input}}-\mu_{B}}{\sqrt{\sigma_{B}^{2}+\varepsilon }}+\beta_{i}$$

where $\mu_{ð}$ and $\sigma_{ð}^{2}$ denote the mean and variance of the current mini-batch, respectively. Here, ε is a fixed numerical-stability constant introduced to avoid division instability when the variance becomes very small. In all experiments, ε was fixed to $10^{-5}$, following the Batch Normalization setting used in the implementation. By contrast, $\gamma_{i}$ and $\beta_{i}$ are the standard learnable affine parameters of the BN layer, rather than manually selected fixed constants. They are initialized using the default BatchNorm configuration and optimized jointly with the rest of the network during training.

It should be emphasized that, although the normalization step depends on mini-batch statistics, the temporal importance indicator used in NTAM is not the instantaneous batch statistic itself, but the learnable scaling parameter $\gamma_{i}$ of the BN layer. Since $\gamma_{i}$ is optimized jointly with the network over the entire training process, the temporal weighting mechanism is less directly affected by the fluctuation of an individual mini-batch than a formulation that directly converts raw batch statistics into attention weights. Nevertheless, because NTAM is still built upon Batch Normalization, extremely small batch sizes may indirectly reduce training stability through noisier feature normalization. This should be regarded as a limitation of the current formulation rather than as a resolved issue.

On top of this normalized representation, NTAM further reuses the scaling factor $\gamma_{i}$ as an indicator of temporal importance. Similar to the normalization-based weighting principle in NAM, a larger absolute scaling factor suggests that the corresponding temporal position is more sensitive to dynamic variation in the normalized feature and is therefore more informative for behavior discrimination.

Based on this correspondence, NTAM normalizes the temporal scaling factors to obtain attention weights, which is formulated as Equation (11):

(11) $$\omega_{\alpha }=\frac{|\gamma_{i}|}{\sum_{j=1}^{T}\left|\gamma_{j}\right|},\;i=1,2,\ldots ,T$$

where the numerator denotes the absolute scaling factor at the *i*-th temporal position and the denominator is the sum of absolute scaling factors over all temporal positions, yielding a comparable distribution of attention intensity along the temporal dimension.

After obtaining the temporal attention weights, NTAM applies them to the normalized feature in a position-wise manner to recalibrate the temporal representation and emphasize key dynamic segments. The final attention-weighted output is expressed as Equation (12):

(12) $$M_{i}=\sigma \left(\omega_{\alpha }⊙\mathrm{BN}\left(F_{\mathrm{input}}\right)\right)$$

where ⊙ denotes element-wise multiplication. Through this adaptive reweighting process, temporal segments with stronger discriminative dynamics are amplified, whereas redundant frames and static background interference are suppressed. Compared with the original NAM design, the present work focuses on adapting normalization-based reweighting to temporal dynamic emphasis in a cascaded classroom behavior recognition pipeline under dense and occluded classroom conditions.

Based on this property, we insert NTAM after the temporal convolution branch of SDS3D. At this stage, SDS3D has already completed the initial separation of spatial and temporal information, so temporal recalibration can focus more effectively on dynamic responses that are truly relevant to discrimination.

Overall, SDS3D and NTAM play complementary roles in the back-end recognition network. SDS3D expands the representational space of the network through diverse spatiotemporal structures, whereas NTAM improves the response quality of key dynamic segments through parameter-free temporal reweighting. Together, they constitute the core design of the back-end behavior-recognition module and provide a structural solution that balances efficiency and accuracy for fine-grained student behavior analysis in complex classroom environments.

## 4. Experimental Results

### 4.1. Dataset Description and Experimental Settings

**Figure 4:**
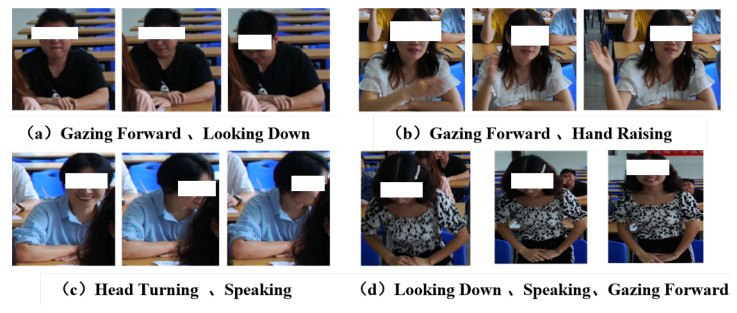
Typical examples of multi-label behavior annotations in classroom scenarios.

To validate the proposed method in real classroom visual perception scenarios, experiments were conducted on the classroom spatiotemporal behavior detection dataset introduced in [34]. This dataset was collected from real classroom videos, with each video lasting approximately 10–60 min and each scene containing about 10–30 students, thus preserving the characteristics of practical teaching environments. The dataset includes classroom videos from primary school, secondary school, and university settings, with variation in student age, seating density, classroom layout, and camera position. Following the standard data format used in spatiotemporal action detection, the raw videos were first segmented into 10 s clips, after which key frames were extracted at 30 fps using ffmpeg. After screening and annotation, the final dataset contained more than 4200 key frames and 51,387 annotated behavior instances. It covers seven typical classroom behaviors: gazing forward, looking down, turning/head rotation, speaking, standing, hand raising, and lying on the desk. To ensure compliance with privacy protection requirements, all raw video data were processed with face blurring and identity dissociation prior to annotation. Only bounding box coordinates and behavior labels were retained for model training, and no personally identifiable information was stored. This procedure aligns with the ethical guidelines for non-intrusive classroom observation. To reflect realistic classroom perception requirements, the dataset presents three major visual challenges that impose stringent demands on algorithmic robustness. First, student distribution is dense and physical occlusion is severe. Multiple students frequently appear in the same frame, overlap between front and rear rows is common, and target scales vary substantially, all of which place heavy demands on the front-end extraction of spatial priors. Second, the dataset contains highly similar fine-grained micro-actions. Typical classroom behaviors such as gazing forward, looking down, and turning are often subtle in magnitude, while their local appearance cues and short-term dynamics are highly confusable, increasing the likelihood of inter-class misclassification. Third, the dataset exhibits complex spatiotemporal multi-label attributes. As illustrated in Figure 4, the dataset adopts dense spatiotemporal bounding-box annotations, and a single student may simultaneously exhibit multiple behavioral attributes at a given moment. This requires the model to jointly parse local spatial and temporal context with sufficient discriminative capacity.

To optimize the proposed model effectively, a decoupled training strategy is adopted. Specifically, the front-end student detector is first trained independently for student localization. After that, the detector outputs are used to generate the RoI-based spatiotemporal inputs for the back-end recognition branch via 3D RoI Align. The back-end recognition model is then trained separately on these localized student regions. In this way, the detector and recognizer are optimized in two stages rather than through end-to-end joint backpropagation, which helps reduce the adverse effect of unstable front-end localization updates on fine-grained behavior recognition.

For the experimental protocol, the dataset was split into training, validation, and test sets at a ratio of 4:1:1. The distribution of instances across categories is summarized in Table 1. To avoid cumulative interference from temporal jitter in front-end detection boxes on back-end spatiotemporal representation learning, the system adopted a decoupled optimization strategy during training. In evaluation, mAP was used as the primary perception metric, while Params, FLOPs, model size, and inference latency were also introduced to assess the system from two complementary perspectives: detection accuracy and edge-deployment efficiency.

### 4.2. Human Target Detection in Dense Scenes

In real classroom environments, student targets are usually densely distributed and frequently occluded. The quality of the candidate regions generated by the front-end detector directly affects the performance of the downstream spatiotemporal behavior recognition branch. Therefore, before evaluating the complete classroom behavior analysis pipeline, it is necessary to separately examine the fundamental detection capability and inference efficiency of the front-end detection module. To this end, the CrowdHuman dataset [35] is adopted as an auxiliary evaluation benchmark. This dataset shares similar detection challenges with classroom scenes in terms of dense human distribution and severe occlusion, and thus provides a unified and standardized basis for comparing different detectors in complex human-centered scenarios. Recent classroom-oriented detection studies have also highlighted that dense distribution, severe occlusion, and limited publicly available benchmarks remain major obstacles to robust front-end perception in student behavior analysis [36].

The objective of this subsection is to evaluate the basic spatial perception capability of front-end detectors under dense-occlusion conditions and to support model selection for the subsequent system design. Different from conventional studies that treat detection performance merely as an accuracy issue, this work emphasizes the dual role of the front-end detector in the overall visual perception pipeline: on the one hand, it provides reliable spatial priors for the downstream spatiotemporal behavior recognition branch; on the other hand, it controls front-end inference latency and prevents the localization stage from becoming a bottleneck of the system. Based on this consideration, comparative experiments are conducted on the CrowdHuman dataset, and the results are reported in Table 2.

The results show that Rep-YOLOv5m achieves a more favorable balance between efficiency and detection performance while maintaining high accuracy. Compared with Faster R-CNN, its parameter scale is substantially smaller, and the inference time is reduced from 291.1 ms to 12.5 ms, indicating that a one-stage detector is more suitable for high-throughput visual perception systems in dense student-target detection scenarios. Furthermore, compared with YOLOv3, YOLOv5s, and YOLOv5m, Rep-YOLOv5m consistently outperforms them in Recall, mAP@0.5, and mAP@0.5:0.95. The single-frame latency of 12.5 ms suggests that this detector can provide higher-quality candidate regions under limited computational budgets.

These results also verify the effectiveness of structural re-parameterization under computation-constrained conditions. Specifically, Rep-YOLOv5m preserves strong representation capability during training through a multi-branch design, while folding these branches into equivalent convolutions during inference, thereby effectively reducing memory access and computational cost. Meanwhile, the RepGhost module adopts additive implicit feature reuse, avoiding the extra memory-copy overhead caused by explicit concatenation. Such a design is particularly suitable for visual sensing pipelines deployed on edge devices or real-time monitoring systems.

From the perspective of classroom behavior recognition, a high-recall front-end detector is of direct importance. The proposed task does not deal with pre-cropped single-person behavior videos, but with multi-target spatiotemporal instances extracted from raw classroom scenes. If a student is missed at the front-end stage, the downstream spatiotemporal network will be unable to obtain the corresponding behavioral representation, and the opportunity for recognition will be lost. Therefore, the high-quality and low-latency spatial priors provided by Rep-YOLOv5m establish a relatively stable input foundation for the subsequent classroom behavior recognition branch.

It should be further noted that the experiments in this subsection only validate the fundamental capability of the front-end detector in standardized dense human scenes, and cannot be directly regarded as its final performance in real classroom behavior analysis tasks. In Section 4.5, the complete Rep-YOLOv5m-SF3D framework will be further evaluated on real classroom video datasets to verify the practical contribution of front-end detector optimization to behavior analysis performance in classroom scenarios.

### 4.3. Ablation of the SDS3D and NTAM Modules

To isolate the impact of the back-end spatiotemporal modeling modules on classroom human activity recognition performance, we ablated the Spatiotemporal Depthwise-Separable 3D module (SDS3D) and the Normalization-Based Temporal Attention Mechanism (NTAM) under the same front-end detection setting. The results are summarized in Table 3. The original SlowFast model already provides a strong baseline on the test set. After introducing either SDS3D or NTAM, the detection accuracy increases further. When both modules are used jointly, mAP reaches 88.83%, which is 3.36% higher than the baseline under the same experimental protocol. This consistent gain suggests that the proposed modules improve the modeling of fine-grained spatiotemporal differences in classroom behaviors.

SDS3D contributes a 1.49% mAP gain over the baseline. Although the factorized convolution structure introduces a slight increase in FLOPs, its strategy of replacing standard 3D convolution with diverse factorized spatiotemporal representations substantially strengthens the network’s ability to characterize spatial posture and temporal dynamics independently. For low-amplitude actions that are highly confusable in classroom scenarios, this structural enhancement expands the feature solution space and improves the clarity of inter-class decision boundaries.

By contrast, NTAM contributes a 1.81% mAP gain in the standalone ablation. As a parameter-free dynamic calibration mechanism, NTAM avoids explicit mapping layers and directly reuses normalization scale factors to adaptively reweight key temporal features in the Fast pathway. Because the effective discriminative cues of classroom behaviors are often concentrated in only a few critical moments, NTAM suppresses static background redundancy with high precision while maintaining a lightweight footprint and marginal computational overhead.

As summarized in Table 4, the module-level improvements exhibit clear physical meaning at the per-class level. The most pronounced change appears in the speaking category. With parameter-free operation, NTAM substantially amplifies the Fast pathway’s response to high-frequency subtle cues, increasing the AP of the speaking class, which relies mainly on mouth dynamics, by 13.48%. For the hand raising category, which contains relatively fewer samples, the joint model still achieves stable improvement, indicating that the diverse response paths introduced by SDS3D retain strong representational capacity even under imbalanced and long-tailed data distributions.

Overall, SDS3D and NTAM exhibit clear complementarity. SDS3D strengthens spatiotemporal modeling for complex actions through factorized representations, whereas NTAM suppresses temporal background redundancy through lightweight dynamic calibration. Their joint use achieves an effective trade-off between representational strength and system efficiency on real classroom human activity recognition tasks.

### 4.4. Comparison with Mainstream Behavior Recognition Methods

To further assess the competitiveness of the proposed method on classroom human activity recognition tasks, the SlowFast-3D recognition branch (SF3D) was compared with several representative spatiotemporal models, including SlowOnly, MViT, LSTC, and the original SlowFast model. These baselines were selected to cover different mainstream design paradigms, including single-stream 3D convolutional modeling, Transformer-based video modeling, temporal-context-enhanced modeling, and dual-stream spatiotemporal modeling. Considering the practical objective of classroom visual sensing, this comparison emphasizes not only recognition accuracy but also model scale and deployment feasibility under resource-constrained settings. The results are summarized in Table 5.

For the comparison in Table 5, all recognition backbones, including SlowOnly, MViT, LSTC, SlowFast, and the proposed SF3D, were initialized from pre-trained weights and then fine-tuned on the same training/validation/test split of the classroom dataset. To ensure a fair comparison, all methods were evaluated under the same target task and unified evaluation protocol. The main training settings were kept consistent across the compared methods as much as permitted by the architectural implementations. Specifically, stochastic gradient descent (SGD) was used as the optimizer, with a weight decay of 0.00001, an initial learning rate of 0.0125, a total of 30 training epochs, and a batch size of 4.

As summarized in Table 5, SF3D achieves the highest mAP, reaching 88.83%, and thus outperforms all competing methods. Relative to the SlowOnly series, the dual-stream architecture of SF3D yields stronger spatiotemporal modeling depth, which supports the view that single-stream models are less capable of separating fine-grained feature differences in complex classroom motion streams. Even when compared with the Transformer-based MViT, SF3D maintains superior recognition accuracy, indicating that under the current dataset scale and task constraints, convolution-based dual-stream modeling with strong inductive bias remains well matched to the domain.

The most informative comparison is against the high-accuracy baseline LSTC. Although LSTC achieves strong accuracy, its model size reaches 274.23 MB and its parameter count reaches 71.8 M, resulting in a substantially higher deployment cost. In contrast, SF3D surpasses LSTC by 2.05% in mAP while reducing model size to 151.46 MB (−46.8%) and Params to 38.17 M (−44.8%). This indicates that the proposed method does not trade scale for accuracy through brute-force expansion, but instead achieves a more effective balance between precision and complexity through structural design. This balance is directly relevant to deployment in real classroom environments.

From an implementation perspective, the performance of RepYOLOv5-SF3D arises from the coordinated interaction of three design components. The dual-stream backbone jointly captures stable semantics and instantaneous dynamics. SDS3D and NTAM improve discrimination for subtle actions. The lightweight design strategy keeps overall overhead within a practical engineering range. These results indicate that the system achieves high recognition accuracy while retaining favorable deployability and scalability, making it suitable for real classroom sensing environments that require high energy efficiency. It should also be noted that the comparison in Table 5 is intended to provide a representative evaluation under classroom-oriented deployment constraints rather than an exhaustive benchmark against all recently proposed large-scale spatiotemporal models.

### 4.5. Gain Analysis of Front-End Priors for the Overall Cascaded Behavior Detection Pipeline

Section 4.2, Section 4.3 and Section 4.4 have separately validated, at the module level, the spatial localization capability of the front-end RepYOLOv5m detector and the spatiotemporal modeling advantages of the back-end SF3D module. However, in real vision-sensor-based scenarios, classroom behavior detection is a visual perception task that depends heavily on the effective coordination between the front-end detection stage and the back-end recognition stage. In the original SlowFast baseline, Faster R-CNN is adopted as the front-end detector. As a computationally heavy two-stage detection framework, it tends to incur substantial inference latency and is more susceptible to missed detections when processing dense and heavily occluded classroom surveillance streams, thereby reducing the completeness of the spatial priors available to the downstream behavior recognition branch. From the perspective of full video-input processing, this subsection aims to evaluate the overall gains of the complete behavior detection pipeline after replacing the original Faster R-CNN with the lightweight RepYOLOv5m. In the experiments, the confidence threshold for object detection was strictly set to 0.8, while the confidence threshold for behavior detection was set to 0.5.

As summarized in Table 6, the number of valid student instances captured in six real classroom videos was compared under different student detectors. RepYOLOv5m consistently detects more student targets. For example, in Video 4, the number of detected students increases from 9 to 15. In dense multi-target scenarios, such high front-end Recall prevents the loss of behavior instances at the localization stage and provides more complete support for downstream action recognition.

To meet the timing requirements of real classroom monitoring, Table 7 further compares the time consumption of different detectors at each stage. Using RepYOLOv5m not only reduces the latency of the object detection stage itself, but also propagates this speedup to the full pipeline, resulting in a consistent reduction in total video behavior detection time. This validates that a lightweight one-stage detector can reduce overall pipeline latency for classroom behavior analysis at the system level.

**Figure 5:**
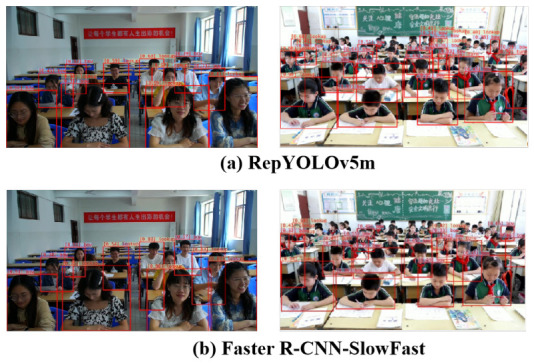
Qualitative comparison of different front-end detectors on the same classroom video segment.

As illustrated in Figure 5, qualitative comparison on the same video segment further supports this conclusion. Faster R-CNN exhibits relatively severe missed detections. Such deficient spatial priors may adversely affect the extraction of spatiotemporal features by the SlowFast network and ultimately degrade behavior recognition accuracy. In contrast, RepYOLOv5m localizes student targets more accurately and maintains favorable detection quality even under severe rear-row occlusion. Taken together, the quantitative and qualitative results indicate that replacing the front end with RepYOLOv5m effectively improves the precision and stability of the overall classroom behavior detection system.

## 5. Discussion and Practical Applications

### 5.1. Analysis of Method Effectiveness in Classroom Visual Sensing Scenarios

The experimental results presented in Section 4 demonstrate that the proposed RepYOLOv5-SF3D cascaded visual perception pipeline effectively addresses the complex challenges encountered in real classroom scenarios, such as dense student distribution, frequent front-to-back occlusion, and low-amplitude behavioral motion. With an mAP of 88.83% on the real classroom video dataset, the pipeline shows robust detection performance in these challenging environments. At the front end, RepYOLOv5m serves as the student detector/spatial-prior node, providing stable student proposals and mitigating the impact of missed detections on subsequent behavior recognition. At the back end, the SlowFast-3D recognition branch (SF3D) improves spatiotemporal representation and temporal dynamic focusing through the Spatiotemporal Depthwise-Separable 3D module (SDS3D) and the Normalization-Based Temporal Attention Mechanism (NTAM), respectively. These enhancements are especially beneficial for behavior categories that rely on short-term local motion cues, reinforcing our hypothesis that this spatiotemporal modeling approach is well-suited for fine-grained classroom behavior analysis. Thus, the contributions of this work extend beyond accuracy improvements; it also establishes a robust classroom visual perception pipeline capable of handling dense targets, subtle local actions, and continuous video analysis within a unified framework.

Further analysis of class-wise results reveals that false positives are most concentrated in several fine-grained categories with visually similar patterns, including speaking, turning/head rotation, and hand raising. These behaviors exhibit lower average precision (AP) values compared to more visually distinct behaviors, such as standing or lying on the desk, indicating their higher susceptibility to recognition errors. Specifically, turning/head rotation involves temporally continuous subtle pose variations, while speaking often depends on weak facial or upper-body motion cues, which are easily degraded under low-resolution or occluded conditions. Although hand raising has improved following model enhancements, partial arm visibility and incomplete local motion still lead to false positives. These findings suggest that the recognition errors are primarily associated with subtle motion ambiguities and insufficient local discriminative cues, particularly in dense classroom environments. Future work will focus on refining motion modeling, enhancing temporal representations, and integrating multimodal fusion to further improve system reliability and reduce errors.

Additionally, NTAM reuses the scale factors from Batch Normalization to generate temporal attention weights. In video recognition tasks with GPU memory constraints, training often requires small batch sizes, which can cause instability in per-batch statistics. NTAM addresses this challenge by relying on the learned BN scale parameters rather than instantaneous batch variance. These parameters are learned and aggregated across training iterations, providing stable and robust indicators for temporal importance even with small batch sizes. This design allows NTAM to effectively emphasize informative temporal segments without adding extra learnable parameters, as demonstrated by the consistent improvements observed in both module-level ablation and per-class AP in our experiments.

### 5.2. Lightweight Design and Engineering Value for Edge Deployment

Beyond recognition performance, the proposed method also carries clear engineering value in terms of system efficiency. For real-time visual perception systems in campus environments, an algorithm must provide reliable recognition while maintaining low inference latency, moderate model scale, and stable long-term operation. The experimental results show that the front-end RepYOLOv5m achieves a single-frame inference time of 12.5 ms, enabling it to provide low-latency spatial priors for the subsequent recognition stage. The back-end SF3D maintains a model size of 151.46 MB and 38.17 M Params, which controls deployment cost while preserving high detection accuracy. These properties indicate that the proposed visual perception pipeline has clear potential for deployment on resource-constrained edge devices, including campus monitoring nodes, edge computing terminals, and lightweight video-analysis equipment. In such settings, it supports long-duration and low-latency analysis of classroom videos and can provide technical support for classroom participation assessment, behavior statistics, and pedagogical feedback. From this perspective, the framework demonstrates favorable real-time inference viability for practical deployment.

### 5.3. Practical Significance and Future Extension Directions

From an application perspective, the proposed method offers a feasible technical route for classroom behavior analysis in smart education scenarios. By continuously detecting student behaviors during class, the system can provide teachers with more objective classroom feedback and also generate data support for instructional-process analysis and pedagogical evaluation. Compared with traditional approaches that rely on manual observation, automated analysis based on visual sensors offers clear advantages in coverage, processing efficiency, and sustained operation, while enabling continuous and non-intrusive monitoring in real-world classrooms.

That said, the current framework still exhibits certain limitations in practical deployment. Although the adopted dataset includes classroom videos from primary school, secondary school, and university settings, with variation in seating density, classroom layout, and camera position, real classroom environments remain highly diverse and complex. Classroom videos often contain severe dense occlusion, viewpoint variation, and complex interactions, all of which remain a non-trivial challenge for behavior analysis based solely on visual input. In addition, some behavior categories, such as speaking, may still be susceptible to visual ambiguity when only subtle facial or upper-body motion is available. These issues suggest that although the current system demonstrates favorable accuracy-efficiency balance, further refinement is still warranted for broader real-world pedagogical deployment.

It should also be noted that the classroom dataset exhibits a clear long-tailed category distribution. Although the present study reports both overall and per-class performance, it does not incorporate a dedicated imbalance-aware training strategy. More specialized designs, such as class-balanced loss functions or category-aware sampling, may further improve the recognition of minority behaviors and remain an important direction for future work.

Future investigations will be directed towards two directions. Firstly, at the algorithmic level, further work will explore more efficient feature-encoding strategies or lighter spatiotemporal modeling structures, aiming to reduce memory-access overhead during joint spatiotemporal modeling and improve computation efficiency on edge computing devices and edge accelerators. Another promising direction is self-supervised video pretraining, which has recently shown strong potential for improving representation quality while reducing dependence on large-scale manual annotation [43,44]. Secondly, at the system level, the framework can be extended toward multimodal sensing. For example, audio sensors may be incorporated to assist in recognizing behavior categories with visual ambiguity, such as speaking, while hardware-level instruction optimization on specific NPU platforms may further improve system latency and scenario adaptability.

## 6. Conclusions

This paper has proposed a lightweight cascaded video behavior analysis framework for student behavior detection in real classroom visual sensing environments. The proposed method takes front-end student localization as the entry point and back-end spatiotemporal behavior recognition as the core. Through the coordinated design of detection and recognition, it achieves unified modeling and analysis of multi-student behaviors in dense classroom scenes. To improve robustness in complex classroom environments, the front-end RepYOLOv5m detector serves as a spatial-prior module, while a decoupled training strategy reduces the impact of localization instability on back-end spatiotemporal learning.

The experimental results have shown that the proposed method achieves favorable detection performance on the classroom behavior detection dataset and also demonstrates good deployment feasibility in terms of system efficiency. On the real classroom dataset, the proposed RepYOLOv5-SF3D framework achieves a mean average precision of 88.83%, outperforming the baseline SlowFast model by 3.36% and surpassing the existing LSTC method by 2.05%, while maintaining a front-end inference latency of 12.5 ms per frame and a total model size of 151.46 MB. The front-end detection mechanism based on structural re-parameterization provides student candidate regions with low latency and high recall. Meanwhile, the back-end spatiotemporal feature factorization strategy and temporal attention mechanism further improve the representation ability for fine-grained spatiotemporal behaviors. In particular, the SDS3D module enhances the separate modeling of spatial structure and temporal dynamics, while the Normalization-Based Temporal Attention Mechanism improves the response to key temporal cues in a parameter-free manner. Overall, the proposed method moves beyond the conventional paradigm of relying excessively on parameter expansion in behavior recognition networks, and achieves a balanced performance in detection accuracy, model scale, and inference efficiency, while maintaining good deployability.

In summary, this work provides an effective solution for video-based student behavior analysis in complex classroom scenarios by taking both accuracy and efficiency into account. It also offers a useful technical reference for subsequent research on edge-oriented visual perception systems for smart education. Therefore, the results indicate that the research objective stated in the Introduction has been achieved, and that the proposed framework effectively addresses the problem of fine-grained student behavior recognition in complex classroom scenes under practical deployment constraints.

## Figures and Tables

**Table 1:** Behavior categories and annotation counts in the classroom behavior detection dataset.

Behavior Category	Annotation Definition	Number of Instances
Gazing Forward/head up	The student faces forward toward the blackboard.	26,131
Looking Down	The student looks downward toward the desk surface.	10,780
Head/Body Turning	The student exhibits a noticeable head or body turn.	5419
Lying on the Desk	The student’s head is close to or resting on the desk.	2010
Speaking	The student is speaking, chatting, or reading aloud.	2909
Standing	The student is standing up or sitting down.	1585
Hand Raising	The student raises one hand upward.	2551
Total	–	51,387

**Table 2:** Comparison of different object detectors.

Model	Params ($10^{6}$)	Recall (%)	Precision (%)	mAP@0.5 (%)	mAP@0.5:0.95 (%)	Inference Time (ms)
Faster R-CNN [37]	186.0	72.06	71.66	72.50	42.60	291.1
YOLOv3 [38]	61.53	85.06	85.25	81.50	55.20	69.0
YOLOv5s [39]	**7.02**	68.51	84.94	79.46	50.37	**10.3**
YOLOv5m [39]	20.88	87.51	86.65	82.10	54.78	14.7
Rep-YOLOv5m	16.67	**89.30**	**86.95**	**83.64**	**55.93**	12.5

**Table 3:** Ablation results for behavior recognition.

SlowFast	SDS3D	NTAM	Parameters/$10^{6}$	Model Size/MB	FLOPs/G	mAP/%
✔			**33.66**	**128.637**	**40.62**	85.47
✔	✔		38.17	151.462	45.87	86.96
✔		✔	**33.66**	128.639	40.63	87.28
✔	✔	✔	38.17	151.464	45.88	**88.83**

**Table 4:** Per-class detection performance before and after module improvement.

Model	Head Up	Looking Down	Head/Body Turning	Speaking	Standing	Hand Raising	Lying on the Desk
SlowFast	95.56	89.09	81.13	62.04	91.07	85.40	93.98
+SDS3D	95.30	89.97	81.67	70.50	92.96	81.89	**96.43**
+NTAM	**95.68**	91.34	79.58	73.05	91.15	85.76	94.37
+SF3D	95.61	**91.95**	**82.02**	**75.52**	**94.22**	**86.32**	96.15

**Table 5:** Performance comparison with representative spatiotemporal behavior recognition methods.

Model	Model Size/MB	Parameters/$10^{6}$	mAP/%
SlowOnly (3D ResNet 50) [40]	**121.50**	**31.8**	75.38
SlowOnly (3D ResNet 101) [40]	194.15	50.8	82.84
MViT [41]	138.51	36.3	74.48
LSTC [42]	274.23	71.8	86.78
SlowFast	128.64	33.66	85.47
SF3D	151.46	38.17	**88.83**

**Table 6:** Comparison of valid student instances captured under different front-end models.

Classroom Video ID	Number of Key Frames	Faster R-CNN	RepYOLOv5m
1	86	10	11
2	87	17	19
3	86	9	10
4	105	9	15
5	86	10	13
6	105	10	14

**Table 7:** Time comparison for end-to-end detection and recognition.

Video ID	Number of Key Frames	Object Detection Time (s)		Video Behavior Detection Time (s)	
		Faster R-CNN	RepYOLOv5m	Faster R-CNN	RepYOLOv5m
1	86	6.48	3.59	17.71	14.90
2	87	8.05	5.28	19.30	16.53
3	86	6.45	3.44	17.66	14.64
4	105	9.52	6.19	23.17	19.89
5	86	6.46	3.48	17.63	14.67
6	105	9.21	6.04	22.18	19.52

## Data Availability

The data presented in this study are available from the https://github.com/zwb146722/Face_datasets.git, (accessed on 5 March 2026).
